# Association Between Telemedicine Adoption and Physician Job Satisfaction: Cross-Sectional Study

**DOI:** 10.2196/82285

**Published:** 2026-03-31

**Authors:** Xiaohui Zhai, Zhongliang Zhou, Zhichao Wang, Guanping Liu, Peter C Coyte

**Affiliations:** 1 School of Public Health, Health Science Center, Xi’an Jiaotong University, Xi’an, Shaanxi, China; 2 School of Public Policy and Administration, Xi’an Jiaotong University, No. 28 Xianning West Road, Xi’an, Shaanxi, 710049, China, 86 18291498261; 3 Institute of Health Policy, Management & Evaluation, Dalla Lana School of Public Health, University of Toronto, Toronto, ON, Canada

**Keywords:** telemedicine, job satisfaction, technology adoption, cross-sectional study, physician patient relationship

## Abstract

**Background:**

Telemedicine has expanded rapidly in recent years, with particularly pronounced growth following the COVID-19 pandemic. By improving access to care and offering greater flexibility in service delivery, it has become an important component of health care. Although the benefits of telemedicine for patients are well documented, its effects on physician job satisfaction remain insufficiently understood. Given the importance of job satisfaction for workforce stability, physician well-being, and quality of care, further examination of how telemedicine affects physician job satisfaction is warranted.

**Objective:**

This study aims to examine the association between telemedicine adoption and physician job satisfaction and to assess whether the physician-patient relationship mediates this association.

**Methods:**

A cross-sectional survey was conducted among health care professionals in Xi’an, China. Data were collected between November 7 and December 8, 2023, via an online questionnaire administered using the REDCap (Research Electronic Data Capture; Vanderbilt University) platform. A total of 12,052 physicians were included in the analysis. Physician job satisfaction was measured using a validated 6-point Likert scale. Telemedicine adoption was assessed through self-report. A partial proportional odds model was used to examine the association between telemedicine adoption and job satisfaction, adjusting for a comprehensive set of potential confounders. Additionally, the Karlson-Holm-Breen (KHB) decomposition method was used to explore the mediating role of physician-patient relationship quality in this association.

**Results:**

Among 12,052 surveyed physicians, 1642 (13.62%) reported adopting telemedicine, whereas 10,410 (86.38%) did not. After adjusting for demographic characteristics, work-related factors, psychological factors, and physician-patient relationship, telemedicine adoption was significantly associated with higher job satisfaction (odds ratio [OR] 1.17, 95% CI 1.05‐1.30). Findings were robust across multiple sensitivity analyses. Subgroup analyses indicated that the association did not vary across physician subgroups, and no significant interaction effects were observed. Mediation analysis revealed a total effect of telemedicine on job satisfaction of 0.33 (95% CI 0.17‐0.50), with an indirect effect of 0.10 (95% CI 0.07‐0.13) through improved physician-patient relationships, accounting for 30.30% of the total effect.

**Conclusions:**

These findings suggest that telemedicine adoption is positively associated with physician job satisfaction, partially mediated by the physician-patient relationship. Policies should promote telemedicine adoption while prioritizing platform designs that support effective physician-patient interactions to enhance provider well-being and care outcomes.

## Introduction

Telemedicine refers to the provision of health care services over a distance using information and communication technologies, enabling remote diagnosis, consultation, treatment, and patient monitoring without the need for physical co-location [1]. Telemedicine has demonstrated significant potential to address longstanding challenges in health care systems. It effectively mitigates barriers such as limited access to care in rural and underserved populations, inefficiencies caused by overcrowded facilities and prolonged patient wait times, uneven distribution of health care resources, and escalating costs associated with traditional in-person care [2]. Globally, the use of telemedicine has increased significantly, especially following the COVID-19 pandemic [3]. According to a World Health Organization (WHO) survey, by 2022, over 70% of member states in the WHO European Region had implemented some form of telemedicine [4].

Telemedicine has become increasingly integrated into diverse clinical contexts, including primary care [5], chronic disease management [6], mental health services [7,8], and specialist consultations [9]. For physicians, this development has significantly altered traditional modes of clinical work. Remote consultations alter the pace and structure of daily workflows, reduce face-to-face interactions, and often require adaptation to new technologies [10]. Clinical decision-making is increasingly supported by digital tools [11], and communication with both patients and colleagues often takes place via electronic platforms.

These shifts have important implications for physicians’ work experience [12], particularly in relation to job satisfaction. Job satisfaction is widely recognized as a critical component of physician well-being and a key determinant of health care system performance. Higher levels of physician satisfaction are consistently associated with improved clinical performance [13,14], lower turnover intentions [15,16], reduced burnout [17], and better patient care [18]. Nevertheless, empirical evidence on the association between telemedicine and physicians’ job satisfaction remains mixed. In Australia, a large national survey of over 7000 physicians demonstrated that those using digital health technologies were approximately 14 percentage points more likely to report higher job satisfaction versus nonusers [19]. Similarly, a US study found that the use of specific telemedicine modalities, particularly video consultations and electronic health record (EHR) integrated platforms, was positively associated with higher physician job satisfaction [20]. However, evidence from a large ambulatory cohort indicated potential drawbacks, as higher telemedicine intensity was associated with increased after-hours EHR workload, which may negatively affect job satisfaction and work-life balance [21]. These divergent findings highlight the need for further investigation into the relationship between telemedicine adoption and physicians’ overall job satisfaction.

Although prior studies have explored the effects of telemedicine on physician job satisfaction, limited attention has been paid to whether this relationship varies across different physician subgroups. Given the substantial variation in physicians’ demographic and professional characteristics, it is plausible that telemedicine may not affect all physicians in the same way [22]. Examining these potential subgroup differences can improve our understanding of how telemedicine influences physician satisfaction and help inform more targeted and effective implementation strategies.

Moreover, few studies have examined the mechanisms underlying the relationship between telemedicine adoption and physician job satisfaction. One potential mediator is the physician-patient relationship. Telemedicine transforms how doctors and patients communicate by enabling clinical interactions to occur on virtual platforms. The adoption of telemedicine can modify key aspects of clinical communication, enabling more flexible information exchange, reduced time burden, and more efficient clarification and follow-up of clinical issues [23]. Such improvements can strengthen the quality of physician-patient interactions and foster a more positive relationship. In turn, this improved relationship is plausibly linked to physicians’ job satisfaction, according to Relational Job Design Theory [24], which posits that more frequent, prolonged, and meaningful interactions with beneficiaries strengthen employees’ perceptions of their positive impact and affective commitment, thereby increasing their motivation to make a prosocial difference. For physicians, stronger prosocial motivation is likely to enhance their engagement in patient care and reinforce their sense of competence and social worth, both of which are strongly linked to higher job satisfaction [25,26]. Taken together, these insights suggest that the physician-patient relationship may serve as a key pathway linking telemedicine adoption to physicians’ job satisfaction.

Given the increasing integration of telemedicine into routine clinical practice, understanding its impact on physician well-being has become increasingly important. This study aims to evaluate the impact of telemedicine adoption on physicians’ job satisfaction and to examine whether this association varies across different physician subgroups. Moreover, the study explores the underlying mechanisms of this relationship by assessing the mediating role of the physician-patient relationship. The findings are expected to provide practical guidance for improving telemedicine implementation and enhancing physician well-being.

## Methods

### Study Design and Participants

This study used data from a large-scale cross-sectional survey conducted among health care professionals in Xi’an, China. A stratified cluster sampling design based on probability proportional to size was used. In total, 47 hospitals and 59 primary health care facilities were selected. All employees within the selected institutions were invited to participate in the survey. Data were collected between November 7 and December 8, 2023, using an online survey administered through REDCap (Research Electronic Data Capture; version 15.2.1; Vanderbilt University). The questionnaire encompassed multiple domains, including demographic characteristics, health status, work-related experiences, attitudes toward internet-based health care, and perceptions of workplace changes.

**Figure 1.**
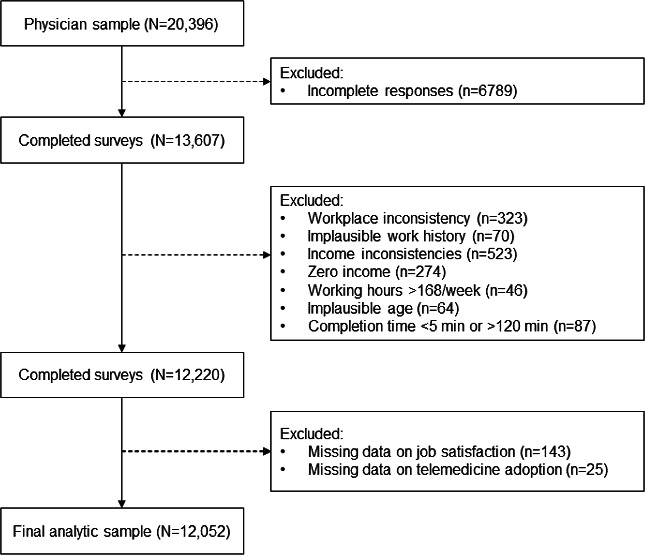
Flowchart of the sample selection process.

For the purposes of this study, only physicians were included in the analysis, as they are the primary providers of telemedicine-based clinical consultations. Physicians were identified based on professional licensure status, including those registered as licensed physicians or assistant physicians in either Western or Traditional Chinese medicine. A total of 20,396 physicians responded to the survey. After excluding incomplete and ineligible responses, 12,052 valid cases remained in the final analytic sample. A detailed overview of the data cleaning and selection process is presented in Figure 1. The reporting of this cross-sectional study adheres to the STROBE (Strengthening the Reporting of Observational Studies in Epidemiology) checklist (Checklist 1).

### Measures

#### Job Satisfaction

Job satisfaction was measured using a single-item statement, “Overall, I am very satisfied with my current job.” Respondents rated their agreement using a 6-point Likert-type scale with the following response options: (1) strongly disagree, (2) disagree, (3) somewhat disagree, (4) somewhat agree, (5) agree, and (6) strongly agree. The use of a single-item measure to capture overall job satisfaction has been validated in prior studies, demonstrating adequate levels of reliability and convergent validity [27,28]. The decision to adopt a 6-point scale, rather than a traditional 5-point or 7-point format, was based on evidence suggesting that even-point Likert scales can reduce central tendency bias by eliminating the neutral option and may provide greater discriminatory power across response categories [29,30].

#### Telemedicine Adoption

The primary independent variable in this study was physicians’ adoption of telemedicine services, which reflects the integration of digital technologies into clinical practice. This variable was measured by asking physicians whether they had provided medical consultations or treatments via internet-based platforms. Responses were binary, with physicians answering “yes” if they had delivered services online and “no” if they had not.

#### Physician-Patient Relationship

To measure the physician-patient relationship from the physician’s viewpoint, a 5-item physician-patient relationship scale was developed based on core relational dimensions identified in prior research and validated instruments such as the Patient-Doctor Relationship Questionnaire (PDRQ-9) [31]. The scale focused on 5 key relational components, including trust, respect, recognition, interpersonal satisfaction, and overall relational quality. Specifically, physicians were asked to rate their perceptions on the following aspects: (1) To what extent do you feel patients respect you? (2) To what extent do you feel patients trust the services you provide? (3) How would you rate the physician-patient relationship? (4) To what extent do you feel patients recognize the value of your work? (5) In most cases, how satisfied are patients with the care you provide? All items were rated on a 5-point Likert scale ranging from 1 (“very poor”) to 5 (“very good”). The average score of the 5 items was calculated to represent the overall level of physician-patient relationship, with higher scores indicating more positive relationships.

To assess the measurement validity of the 5-item physician-patient relationship scale, we conducted an exploratory factor analysis, which yielded a Kaiser-Meyer-Olkin (KMO) value of 0.82 and a significant Bartlett test of sphericity (*χ*²_10_=19,569.89; *P*<.001). All 5 items loaded strongly on the factor (loadings ranged from 0.59 to 0.74). The internal consistency of the scale was good, with a Cronbach α of 0.81.

### Control Variables

We included three sets of control variables in the analysis: demographic, work-related characteristics, and psychological factors. Physician demographics comprised sex (male or female), age (categorized as ≤30, 31‐44, 45‐59, and ≥60 years), marital status (married or unmarried), and educational attainment (categorized as associate degree or below, bachelor’s degree, master’s degree, or doctoral degree).

Work-related characteristics included professional title, hospital type, employment status, medical specialty, managerial position, years in practice, years of service at the current institution, monthly income, and weekly working hours. In China, physicians hold professional titles that indicate their career stage. Professional titles were used as a proxy for career seniority and categorized into 4 levels according to the Chinese medical title system, including no title, junior, mid-level, and senior. As hierarchical levels are defined and routinely applied within the public hospital system in China, and such information was not collected in our data for private hospitals, hospitals were categorized into 4 types, including tertiary, secondary, primary, and private hospitals. Employment status was measured by asking whether physicians held a permanent (tenured) position at their institution. Responses were coded as 1 for permanent employee and 0 for contract employee. Medical specialty was classified according to the physician’s department and included the following categories: clinical, medical technology, general practitioner, and others. The managerial position was coded as a binary variable indicating whether the physician held any administrative or leadership roles within their department, with 1 representing “yes” and 0 representing “no.” Years in practice were measured by asking physicians how many years they have been practicing medicine since completing their training. Years of service at the current institution were assessed by asking physicians how long they had been employed at their current workplace.

Psychological factors were measured using indicators of work stress, depression, and anxiety. Work stress was assessed using “Overall, I feel a high level of work-related stress,” with responses on a 6-point Likert scale ranging from strongly disagree to strongly agree. For analysis, responses were grouped into 3 categories: low stress (combining strongly disagree and disagree), moderate stress (combining somewhat disagree and somewhat agree), and high stress (combining agree and strongly agree). Depression and anxiety were assessed by self-reported clinical diagnosis, with participants indicating whether they have ever been diagnosed with depression or anxiety disorders.

### Statistical Analysis

All statistical analyses were performed using STATA (version 17 MP; StataCorp LLC) and R (version 4.5.0; R Foundation for Statistical Computing). Statistical significance was set at a 2-sided *P*<.05.

Descriptive statistics were first calculated to summarize the characteristics of physicians. Given the skewed distribution of continuous variables, medians and IQRs were reported. Categorical variables were summarized using frequencies and percentages. To compare differences between physicians who provided telemedicine services and those who did not, the Mann-Whitney *U* test was applied for continuous variables, and the chi-square test was used for categorical variables.

Given the ordered nature of the job satisfaction outcome, we initially fitted an ordered logistic regression model to examine the association between telemedicine adoption and physicians’ job satisfaction. The proportional odds assumption was evaluated using the Brant test [32]. The overall test indicated that the assumption was violated (*χ*²_116_=357.04; *P*<.001), although telemedicine adoption met the proportionality requirement (*χ*²_4_=4.16; *P*=.39). The detailed Brant test results are presented in Table S1 in Multimedia Appendix 1. To address these violations and avoid biased estimates, we used a partial proportional odds model, which relaxes the proportional odds constraint for variables that do not meet the assumption while maintaining the constraint for variables that do [33]. The final model included telemedicine adoption as the primary independent variable and adjusted for all control variables, including physicians’ demographic characteristics, work-related factors, psychological factors, and the physician-patient relationship. Subgroup analyses were performed to examine potential variations in the relationship between telemedicine adoption and job satisfaction across all control variables. Continuous variables (eg, years in practice and weekly working hours) were dichotomized at their median values to ensure balanced group sizes for subgroup comparisons.

We used the Karlson-Holm-Breen (KHB) method to examine whether the physician-patient relationship mediated the association between telemedicine adoption and job satisfaction. The KHB approach allows for the decomposition of total effects into direct and indirect components in non-linear probability models [34]. This method effectively corrects for the rescaling problem that arises when comparing coefficients across nested nonlinear models, thereby providing unbiased estimates of mediation effects [35].

We conducted additional analyses to verify the robustness of our main finding. First, we re-estimated the main models using ordinary least squares regression as an alternative specification. Second, we used an alternative measure of job satisfaction based on a 5-point Likert item from the questionnaire, “Overall, I am very satisfied with my current job,” with responses ranging from strongly disagree to strongly agree, to test the consistency of results across different satisfaction measures. Third, we used physicians’ weekly online patient volume as a continuous measure of telemedicine adoption to assess the robustness of our findings.

### Ethical Considerations

This study was approved by the Biomedical Ethics Committee of Xi’an Jiaotong University (XJTUAE2646). Electronic informed consent was obtained from all participants. All responses were anonymous, and no personal identifying information was collected. No compensation was provided to participants.

## Results

### Descriptive Statistics

The basic characteristics of physicians are displayed in Table 1. Among the 12,052 physicians, 1642 (13.62%) reported adopting telemedicine services, while 10,410 (86.38%) did not use telemedicine. The sample comprised 7351 out of 12,052 (61.08%) female physicians. Over half of the participants (6318/12,052; 52.42%) were aged 31-44 years. Most physicians were married. Regarding educational attainment, 5380 out of 12,052 (44.64%) held a bachelor’s degree and 4512 out of 12,052 (37.44%) held a master’s degree. Physicians with mid-level professional titles accounted for 39.17% (4712/12,052) of the sample, followed by those with a junior title (3850/12,052; 32.00%). More than half of the physicians (6765/12,052; 56.20%) were employed at tertiary hospitals. About 65.73% (7282/12,052) of the physicians held permanent positions. Clinicians accounted for 53.71% (6465/12,052) of the sample. The proportion of physicians holding managerial positions was 24.89% (3000/12,052). The median years of practice and years of service at the current institution were 12 and 8, respectively. The median monthly income was 8000 yuan (US $1159.34). The median number of weekly working hours was 50. Physicians who perceived work stress as “high” and “moderate” accounted for 48.14% (5800/12,052) and 42.98% (5179/12,052), respectively. The prevalence of depression and anxiety among participants was 4.32% (521/12,052) and 10.33% (1245/12,052), respectively.

**Table 1.** Characteristics of physicians stratified by telemedicine adoption.

Variables^a^	Total (N=12,052)	Telemedicine adoption		*P* value
		Yes (n=1642)	No (n=10,410)	
Sex, n (%)				<.001
Female	7351 (61.08)	797 (48.60)	6554 (63.05)	
Male	4684 (38.92)	843 (51.40)	3841 (36.95)	
Age (years), n (%)				<.001
≤30	2409 (19.99)	113 (6.88)	2296 (22.06)	
31-44	6318 (52.42)	858 (52.25)	5460 (52.45)	
45-59	3077 (25.53)	612 (37.27)	2465 (23.68)	
≥60	248 (2.06)	59 (3.59)	189 (1.82)	
Marital status, n (%)				<.001
Married	9862 (82.00)	1481 (90.30)	8381 (80.69)	
Unmarried^b^	2165 (18.00)	159 (9.70)	2006 (19.31)	
Education, n (%)				<.001
Associate degree or below	1449 (12.02)	137 (8.34)	1312 (12.60)	
Bachelor’s degree	5380 (44.64)	658 (40.07)	4722 (45.36)	
Master’s degree	4512 (37.44)	615 (37.45)	3897 (37.44)	
Doctoral degree	711 (5.90)	232 (14.13)	479 (4.60)	
Professional titles, n (%)				<.001
No title	266 (2.21)	13 (0.79)	253 (2.43)	
Junior	3850 (32.00)	220 (13.41)	3630 (34.93)	
Mid-level	4712 (39.17)	564 (34.39)	4148 (39.92)	
Senior	3203 (26.62)	843 (51.40)	2360 (22.71)	
Hospital type, n (%)				<.001
Tertiary hospitals	6765 (56.20)	1063 (64.78)	5702 (54.84)	
Secondary hospitals	2242 (18.62)	203 (12.37)	2039 (19.61)	
Primary hospitals	2233 (18.55)	163 (9.93)	2070 (19.91)	
Private hospitals	798 (6.63)	212 (12.92)	586 (5.64)	
Employment status, n (%)				<.001
Permanent employee	7282 (65.73)	1067 (75.46)	6215 (64.30)	
Contract employee	3797 (34.27)	347 (24.54)	3450 (35.70)	
Medical specialty, n (%)				<.001
Clinical	6465 (53.71)	1079 (65.75)	5386 (51.81)	
Medical technology	376 (3.12)	28 (1.71)	348 (3.35)	
General practitioner	1224 (10.17)	117 (7.13)	1107 (10.65)	
Others	3971 (32.99)	417 (25.41)	3554 (34.19)	
Managerial position, n (%)				<.001
No	9052 (75.11)	1033 (62.91)	8019 (77.03)	
Yes	3000 (24.89)	609 (37.09)	2391 (22.97)	
Years in practice, median (IQR)	12 (6‐21)	17 (10‐26)	11 (5‐20)	<.001
Years of service at the current institution, median (IQR)	8 (3-15)	12 (6‐20)	7 (3-14)	<.001
Monthly income^c^, median (IQR)	8000 (5000‐10,000)	10,000 (7000‐15,000)	7500 (5000‐10,000)	<.001
Weekly working hours, median (IQR)	50 (40‐60)	50 (45‐60)	50 (40‐60)	<.001
Work stress, n (%)				<.001
Low	1070 (8.88)	132 (8.04)	938 (9.01)	
Moderate	5179 (42.98)	633 (38.57)	4546 (43.68)	
High	5800 (48.14)	876 (53.38)	4924 (47.31)	
Depression, n (%)				.009
No	11,531 (95.68)	1551 (94.46)	9980 (95.87)	
Yes	521 (4.32)	91 (5.54)	430 (4.13)	
Anxiety, n (%)				<.001
No	10,807 (89.67)	1423 (86.66)	9384 (90.14)	
Yes	1245 (10.33)	219 (13.34)	1026 (9.86)	
Physician-patient relationship, median (IQR)	3.8 (3.4‐4)	3.8 (3.4‐4.2)	3.8 (3.4‐4)	<.001

a Counts are based on valid (nonmissing) responses for each variable.

b Individuals who are single (never married), divorced, or widowed.

c Income is measured in Chinese Yuan (RMB), in 2023, the average exchange rate was approximately US $1=7.08 RMB.

Significant differences were observed between physicians who adopted telemedicine and those who did not across multiple characteristics. Compared to those who did not adopt telemedicine, physicians who adopted telemedicine were more likely to be male, older, married, hold a doctoral degree and senior professional titles, work in tertiary hospitals, be permanent employees, work in clinical departments, possess managerial roles, have longer years of practice and tenure at their current institution, and report higher monthly income (all *P*<.001). Additionally, telemedicine adopters reported higher work stress and were more likely to experience symptoms of depression and anxiety (all *P*<.01).

### Association Between Telemedicine Adoption and Physician Job Satisfaction

We used a partial proportional odds model to estimate the association between telemedicine adoption and job satisfaction among physicians. The telemedicine adoption met the proportional odds assumption, indicating a consistent effect across cumulative logits. As shown in Table 2, telemedicine adoption was positively associated with higher job satisfaction. Specifically, physicians who adopted telemedicine had 17% higher odds of reporting a higher satisfaction level compared with those who did not (odds ratio [OR] 1.17, 95% CI 1.05‐1.30).

**Table 2.** Association between telemedicine adoption and physician job satisfaction.

Variables	Physician job satisfaction, OR^a^ (95% CI)				
	Very dissatisfied	Dissatisfied	Somewhat dissatisfied	Somewhat satisfied	Satisfied
Telemedicine adoption (reference=no)					
Yes	1.17^b^ (1.05-1.30)	1.17^b^ (1.05-1.30)	1.17^b^ (1.05-1.30)	1.17^b^ (1.05-1.30)	1.17^b^ (1.05-1.30)
Sex (reference=female)					
Male	0.70^b^ (0.55-0.90)	0.87 (0.74-1.02)	1.13^c^ (1.02-1.25)	1.13^b^ (1.03-1.23)	1.24^d^ (1.11-1.39)
Age (years), (reference ≤30)					
31-44	0.97 (0.85-1.10)	0.97 (0.85-1.10)	0.97 (0.85-1.10)	0.97 (0.85-1.10)	0.97 (0.85-1.10)
45-59	1.13 (0.78-1.63)	1.08 (0.82-1.42)	0.99 (0.79-1.25)	1.10 (0.88-1.36)	1.26 (1.00-1.59)
≥60	1.45 (0.98-2.14)	1.45 (0.98-2.14)	1.45 (0.98-2.14)	1.45 (0.98-2.14)	1.45 (0.98-2.14)
Marital status (reference=unmarried)					
Married	1.78^d^ (1.33-2.37)	1.40^b^ (1.15-1.71)	1.25^b^ (1.09-1.43)	1.08 (0.96-1.22)	1.05 (0.90-1.23)
Education (reference=associate degree or below)					
Bachelor’s degree	0.80^b^ (0.68-0.93)	0.80^b^ (0.68-0.93)	0.80^b^ (0.68-0.93)	0.80^b^ (0.68-0.93)	0.80^b^ (0.68-0.93)
Master’s degree	0.73^b^ (0.60-0.88)	0.73^b^ (0.60-0.88)	0.73^b^ (0.60-0.88)	0.73^b^ (0.60-0.88)	0.73^b^ (0.60-0.88)
Doctoral degree	0.71^b^ (0.56-0.90)	0.71^b^ (0.56-0.90)	0.71^b^ (0.56-0.90)	0.71^b^ (0.56-0.90)	0.71^b^ (0.56-0.90)
Professional titles (reference=no title)					
Junior	0.80 (0.62-1.03)	0.80 (0.62-1.03)	0.80 (0.62-1.03)	0.80 (0.62-1.03)	0.80 (0.62-1.03)
Mid-level	0.58^d^ (0.45-0.76)	0.58^d^ (0.45-0.76)	0.58^d^ (0.45-0.76)	0.58^d^ (0.45-0.76)	0.58^d^ (0.45-0.76)
Senior	0.60^d^ (0.45-0.79)	0.60^d^ (0.45-0.79)	0.60^d^ (0.45-0.79)	0.60^d^ (0.45-0.79)	0.60^d^ (0.45-0.79)
Hospital type (reference =tertiary hospitals)					
Secondary hospitals	0.83^b^ (0.75-0.93)	0.83^b^ (0.75-0.93)	0.83^b^ (0.75-0.93)	0.83^b^ (0.75-0.93)	0.83^b^ (0.75-0.93)
Primary hospitals	0.71^d^ (0.62-0.81)	0.71^d^ (0.62-0.81)	0.71^d^ (0.62-0.81)	0.71^d^ (0.62-0.81)	0.71^d^ (0.62-0.81)
Private hospitals	4.67e-11^d^ (4.36e-11 to 5.00e-11)	23.41 (0.01-590922.3)	0.48 (0.19-1.20)	1.69 (0.79-3.64)	2.30^c^ (1.14-4.65)
Employment status (reference=contract employee)					
Permanent employee	0.86^d^ (0.79-0.93)	0.86^d^ (0.79-0.93)	0.86^d^ (0.79-0.93)	0.86^d^ (0.79-0.93)	0.86^d^ (0.79-0.93)
Medical specialty (reference=clinical)					
Medical technology	1.25^c^ (1.02-1.53)	1.25^c^ (1.02-1.53)	1.25^c^ (1.02-1.53)	1.25^c^ (1.02-1.53)	1.25^c^ (1.02-1.53)
General practitioner	1.02 (0.89-1.16)	1.02 (0.89-1.16)	1.02 (0.89-1.16)	1.02 (0.89-1.16)	1.02 (0.89-1.16)
Others	1.05 (0.97-1.13)	1.05 (0.97-1.13)	1.05 (0.97-1.13)	1.05 (0.97-1.13)	1.05 (0.97-1.13)
Managerial position (reference=no)					
Yes	1.44^d^ (1.32-1.58)	1.44^d^ (1.32-1.58)	1.44^d^ (1.32-1.58)	1.44^d^ (1.32-1.58)	1.44^d^ (1.32-1.58)
Years in practice	1.00 (0.99-1.01)	1.00 (0.99-1.01)	1.00 (0.99-1.01)	1.00 (0.99-1.01)	1.00 (0.99-1.01)
Years of service at the current institution	0.99^c^ (0.99-1.00)	0.99^c^ (0.99-1.00)	0.99^c^ (0.99-1.00)	0.99^c^ (0.99-1.00)	0.99^c^ (0.99-1.00)
ln(Income)^e^	1.03 (0.99-1.08)	1.03 (0.99-1.08)	1.03 (0.99-1.08)	1.03 (0.99-1.08)	1.03 (0.99-1.08)
Weekly working hours	1.00^d^ (0.99-1.00)	1.00^d^ (0.99-1.00)	1.00^d^ (0.99-1.00)	1.00^d^ (0.991.00)	1.00^d^ (0.99-1.00)
Work stress (reference=low)					
Moderate	4.76^d^ (2.74-8.26)	2.44^d^ (1.78-3.33)	0.73^b^ (0.58-0.91)	0.24^d^ (0.20-0.29)	0.21^d^ (0.18-0.25)
High	1.04 (0.67-1.64)	1.05 (0.79-1.40)	0.57^d^ (0.46-0.72)	0.38^d^ (0.32-0.45)	0.33^d^ (0.28-0.38)
Depression (reference=no)					
Yes	0.49^d^ (0.34-0.71)	0.49^d^ (0.37-0.65)	0.52^d^ (0.41-0.65)	0.73^b^ (0.58-0.92)	1.14 (0.80-1.63)
Anxiety (reference=no)					
Yes	0.42^d^ (0.31-0.58)	0.62^d^ (0.49-0.77)	0.79^b^ (0.67-0.92)	0.66^d^ (0.57-0.77)	0.72^b^ (0.57-0.92)
Physician-patient relationship	3.05^d^ (2.47-3.76)	3.03^d^ (2.61-3.51)	2.79^d^ (2.53-3.08)	3.41^d^ (3.13-3.71)	4.54^d^ (4.06-5.08)

a OR: odds ratio.

b *P*<.01.

c *P*<.05.

d *P*<.001.

e ln(Income) refers to the natural logarithm of monthly income.

In addition to telemedicine adoption, several covariates were significantly associated with job satisfaction, and many exhibited clear nonproportional effects across the satisfaction thresholds. For example, in the lowest satisfaction category, males are less likely to report higher satisfaction levels than females (OR 0.70, 95% CI 0.55‐0.90), whereas at higher thresholds the direction of the association reversed, with males more likely to report higher satisfaction (eg, OR 1.24, 95% CI 1.11‐1.39). Married physicians were more likely to report higher satisfaction than unmarried physicians at the lower thresholds, but the association diminished and was not statistically significant at the higher satisfaction levels. Physicians with moderate stress were more likely to report higher satisfaction at the lower thresholds but were less likely to report higher levels of satisfaction at the higher thresholds. High stress was associated with lower satisfaction at the higher thresholds. Physicians with depressive symptoms were less likely to report higher satisfaction at the lower and middle thresholds, with the association weakening at the highest satisfaction level. Physicians with anxiety were less likely to report higher satisfaction across all thresholds. Better physician-patient relationships were strongly and consistently associated with higher satisfaction across all thresholds, with stronger effects observed at the highest satisfaction level.

For covariates meeting the proportional-odds assumption, the estimated effects were consistent across all satisfaction thresholds. Physicians with higher education and those holding mid-level or senior professional titles exhibited significantly lower job satisfaction. Employment in secondary and primary hospitals was associated with reduced satisfaction relative to tertiary hospitals. Permanent employees reported lower levels of job satisfaction than contract employees (OR 0.86, 95% CI 0.79‐0.93). Physicians in medical technology specialties reported higher satisfaction than those in clinical specialties (OR 1.25, 95% CI 1.02‐1.53). Holding a managerial position showed a positive association with job satisfaction (OR 1.44, 95% CI 1.32‐1.58). Longer tenure was associated with slightly higher odds of reporting lower satisfaction (OR 0.99, 95% CI 0.99‐1.00). Longer weekly working hours were negatively associated with job satisfaction (OR 1.00, 95% CI 0.99‐1.00).

### Subgroup Analyses

**Figure 2.**
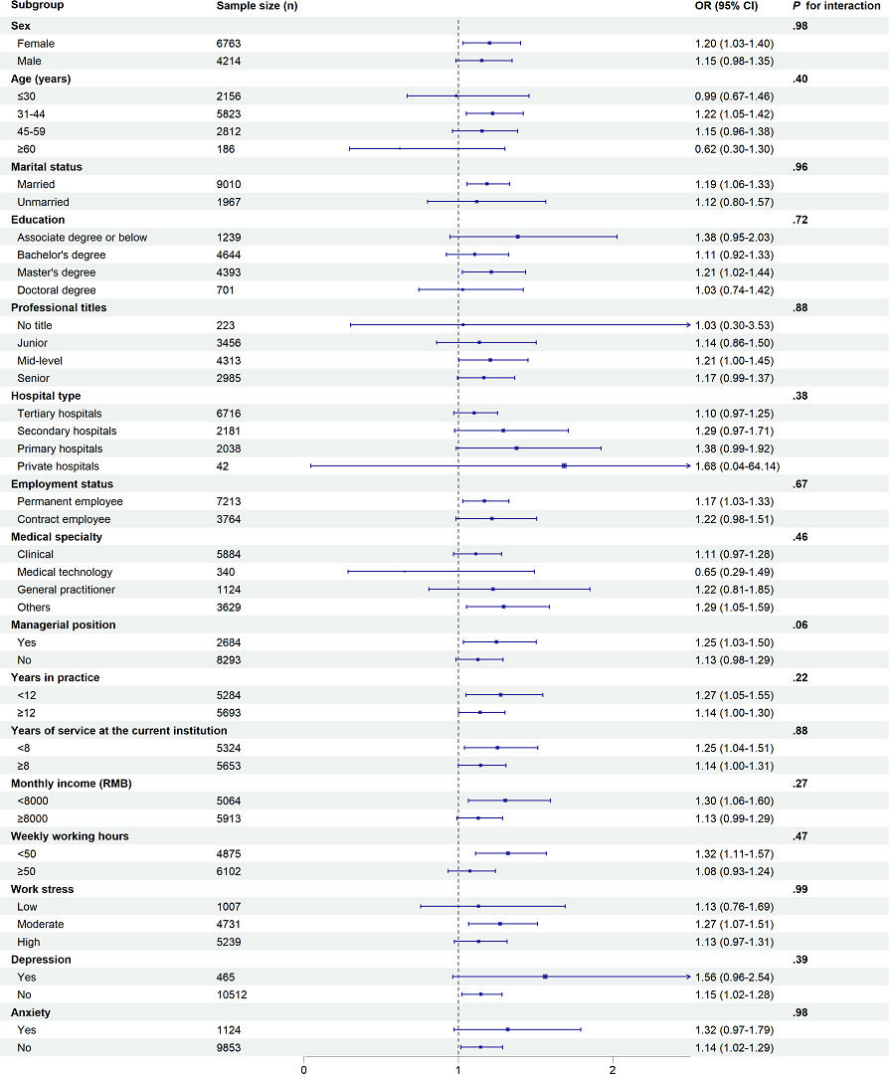
Forest plot of subgroup analyses on the association between telemedicine adoption and physician job satisfaction. Income is measured in Chinese Yuan (RMB). In 2023, the average exchange rate was approximately US $1=7.08 RMB. OR: odds ratio.

Subgroup analyses were conducted to examine whether the association between telemedicine adoption and physician job satisfaction differed by physician characteristics. The association was generally consistent across groups, with no significant interaction effects detected (all *P* for interaction >.05), as illustrated in Figure 2. Notably, the association was statistically significant among female physicians (OR 1.20, 95% CI 1.03‐1.40), those aged 31‐44 years (OR 1.22, 95% CI 1.05‐1.42), married physicians (OR 1.19, 95% CI 1.06‐1.33), those with a master’s degree (OR 1.21, 95% CI 1.02‐1.44), permanent employees (OR 1.17, 95% CI 1.03‐1.33), and physicians in the “others” specialty group (OR 1.29, 95% CI 1.05‐1.59). In addition, significant associations were found among physicians with managerial positions (OR 1.25, 95% CI 1.03‐1.50), those with fewer than 12 years of practice (OR 1.27, 95% CI 1.05‐1.55), those with less than 8 years of service at the current institution (OR 1.25, 95% CI 1.04‐1.51), those with monthly income below 8000 RMB (US $1159.34; OR 1.30, 95% CI 1.06‐1.60), and those working fewer than 50 hours per week (OR 1.32, 95% CI 1.11‐1.57). Finally, telemedicine adoption was positively associated with job satisfaction among physicians reporting moderate work stress (OR 1.27, 95% CI 1.07‐1.51), those without depression (OR 1.15, 95% CI 1.02‐1.28), and those without anxiety symptoms (OR 1.14, 95% CI 1.02‐1.29).

### Mediation Analyses

Table 3 presents the results of the KHB decomposition analysis. The results indicated a significant partial mediation effect. The total effect of telemedicine adoption on job satisfaction was 0.33 (95% CI 0.17-0.50). After accounting for the physician-patient relationship, the direct effect decreased to 0.23 (95% CI 0.07-0.39), while the indirect effect through the physician-patient relationship was 0.10 (95% CI 0.07-0.13). This indirect pathway accounted for 30.30% of the total effect, suggesting that the physician-patient relationship plays an important mediating role between telemedicine adoption and job satisfaction.

**Table 3.** Mediation effect of physician-patient relationship on telemedicine and job satisfaction.

Effect	*β* (95% CI)	*P* value	Proportion of mediating effect
Total effect	.33 (0.17-0.50)	<.001	—^a^
Direct effect	.23 (0.07-0.39)	.006	69.70%
Indirect effect	.10 (0.07-0.13)	<.001	30.30%

a Not available.

### Sensitivity Analysis

The sensitivity analyses supported the robustness of our main findings. First, we re-estimated the models using ordinary least squares regression as a robustness check, and the results remained consistent with the original models in terms of direction and statistical significance (Table S2 in Multimedia Appendix 1). Second, when job satisfaction was measured using an alternative 5-point Likert item, the association between telemedicine adoption and job satisfaction remained robust (Table S3 in Multimedia Appendix 1). Third, we replaced the binary indicator of telemedicine adoption with a continuous measure reflecting the number of patients a physician consulted online per week. The results remained robust, further supporting the validity of our main findings (Table S4 in Multimedia Appendix 1).

## Discussion

### Principal Findings

In this cross-sectional study of physicians, telemedicine adoption was significantly associated with higher levels of job satisfaction. Physicians who adopted telemedicine reported higher levels of job satisfaction compared with those who did not, and this association remained robust across multiple sensitivity analyses. Mediation analysis further suggested that the physician-patient relationship partially explained this association. These findings contribute to a growing body of literature examining how digital health technologies affect physicians’ work experiences and underscore the importance of preserving strong interpersonal relationships in virtual care settings.

### Comparison With Prior Work

The telemedicine adoption rate in our study was 13.62%, which appears relatively low compared to telemedicine adoption rates reported in other countries, where physician uptake during the COVID-19 pandemic ranged from approximately 30% to 87% [36-38]. This difference may be partly attributable to variations in the measurement of telemedicine adoption. In our study, adoption was defined specifically as whether physicians had provided medical consultations or treatments via internet-based platforms, whereas some international studies use broader definitions of telemedicine that include not only direct patient consultations but also activities such as exchanging referrals with other providers, ordering and reviewing diagnostic tests, or other forms of remote care, thereby yielding higher reported rates. Additionally, regional differences and health care system characteristics in Xi’an and broader China may play a role.

Consistent with previous research, our study found that telemedicine adoption is positively associated with physician job satisfaction [19,20]. This association may reflect several potential advantages of telehealth services that directly influence physicians’ work experiences. Previous studies have found that telemedicine enhances scheduling flexibility and work-life balance among physicians [19], reduces administrative burdens [39], and increases practice productivity [40], all of which are closely linked to lower stress and higher job satisfaction. Additionally, telemedicine may support more efficient patient follow-up and continuity of care, particularly in primary care [41] and chronic disease management settings [42,43], thereby improving the clinical workflow. Taken together, these features may contribute to a more sustainable and fulfilling clinical practice.

Subgroup analyses demonstrated that the positive association between telemedicine adoption and physician job satisfaction was generally consistent across various demographic and professional groups, with no significant interaction effects observed. These findings imply that telemedicine adoption is broadly beneficial for physician job satisfaction, regardless of demographic or work-related characteristics. Nonetheless, the significant associations within certain subgroups indicate that the effect may be more detectable among physicians sharing specific characteristics.

The mediation analysis revealed a significant partial mediating effect, indicating that telemedicine adoption is associated with a more positive physician-patient relationship, which in turn contributes to higher job satisfaction. Specifically, physicians who used telemedicine tended to report higher physician-patient relationship scores. Empirical studies similarly show that telemedicine enables more frequent, timely, and continuous contact, which strengthens physicians’ sense of connection and familiarity with their patients, thereby fostering a more trusting and collaborative physician-patient relationship [44,45]. Such positive relational experiences have been associated with higher job satisfaction [46], partly because strong physician-patient relationships enhance physicians’ sense of professional efficacy, reduce workplace stress, and reinforce the meaningfulness of their clinical work. Overall, these findings suggest that improvements in physician-patient relationships represent an important pathway through which telemedicine may enhance physicians’ job satisfaction.

Building on these findings, several policy implications can be drawn. Health care systems should facilitate telemedicine adoption through targeted training and resource allocation tailored to physicians’ diverse needs. The design of telemedicine platforms should explicitly incorporate features aimed at improving the quality of the physician-patient relationship. Additionally, workload management strategies and support mechanisms are necessary to mitigate potential burnout associated with telemedicine use.

### Limitations

Several limitations of this study warrant careful consideration. First, the cross-sectional design inherently restricts the ability to draw causal inferences regarding the relationship between telemedicine use and physicians’ job satisfaction. Longitudinal studies are needed to establish temporal dynamics and causal pathways. Second, job satisfaction was assessed using a single-item measure. While this approach has been validated in previous research and is suitable for large-scale surveys with space constraints, multi-item instruments could offer a more detailed understanding of satisfaction dimensions. Third, the survey measured telemedicine use based on whether physicians reported using telemedicine for diagnosis or treatment, without distinguishing specific modalities such as video consultations, asynchronous messaging, or phone visits. Future studies with more detailed information on specific telemedicine modalities could clarify how different forms of telemedicine influence physicians’ job satisfaction. Finally, although a stratified probability proportional to size sampling approach was used to enhance representativeness, the sample is limited to physicians within Xi’an, a specific urban context in China. This geographic concentration may limit the external validity of findings, as organizational cultures, telemedicine infrastructure, and health care policies vary substantially across regions and countries.

### Conclusions

In this cross-sectional study, telemedicine adoption was positively associated with physician job satisfaction. This association was partially mediated by the physician-patient relationship, suggesting that enhanced communication and connection through telemedicine contribute to higher satisfaction. To optimize these benefits, physicians need to use telemedicine appropriately to balance its benefits and challenges. Meanwhile, health care institutions should provide targeted support, including communication training and stress management, to promote effective and sustainable telemedicine practice.

## Supplementary material

10.2196/82285 Multimedia Appendix 1 Results of Brant test and robustness checks.

10.2196/82285 Checklist 1 The STROBE checklist.

## Abbreviations

EHR: electronic health record
KHB: Karlson-Holm-Breen
KMO: Kaiser-Meyer-Olkin
OR: odds ratio
PDRQ-9: Patient-Doctor Relationship Questionnaire-9 item
REDCap: Research Electronic Data Capture
STROBE: Strengthening the Reporting of Observational Studies in Epidemiology
WHO: World Health Organization

## References

[1] World Health Organization (2010). Telemedicine: Opportunities and Developments in Member States: Report on the Second Global Survey on eHealth.

[2] Bashshur RL, Shannon GW, Smith BR (2014). The empirical foundations of telemedicine interventions for chronic disease management. Telemed J E Health.

[3] Suran M (2022). Increased use of medicare telehealth during the pandemic. JAMA.

[4] Gullslett MK, Ronchi E, Lundberg L (2024). Telehealth development in the WHO European region: results from a quantitative survey and insights from Norway. Int J Med Inform.

[5] Orozco-Beltran D, Sánchez-Molla M, Sanchez JJ, Mira JJ, ValCrònic Research Group (2017). Telemedicine in primary care for patients with chronic conditions: the ValCrònic quasi-experimental study. J Med Internet Res.

[6] Lewinski AA, Walsh C, Rushton S (2022). Telehealth for the longitudinal management of chronic conditions: systematic review. J Med Internet Res.

[7] Fortney JC, Bauer AM, Cerimele JM (2021). Comparison of teleintegrated care and telereferral care for treating complex psychiatric disorders in primary care: a pragmatic randomized comparative effectiveness trial. JAMA Psychiatry.

[8] Wu M, Li C, Hu T (2024). Effectiveness of telecare interventions on depression symptoms among older adults: systematic review and meta-analysis. JMIR Mhealth Uhealth.

[9] Di Cerbo A, Morales-Medina JC, Palmieri B, Iannitti T (2015). Narrative review of telemedicine consultation in medical practice. Patient Prefer Adherence.

[10] Greenhalgh T, Rosen R, Shaw SE (2021). Planning and evaluating remote consultation services: a new conceptual framework incorporating complexity and practical ethics. Front Digit Health.

[11] Shah KM, Shah MM, Shah IP, Patel HH (2024). The role of technology in enhancing clinical decision-making for health professiona. Front Health Inform.

[12] Kaihlanen AM, Laukka E, Nadav J (2023). The effects of digitalisation on health and social care work: a qualitative descriptive study of the perceptions of professionals and managers. BMC Health Serv Res.

[13] Scheepers RA (2017). Physicians’ professional performance: an occupational health psychology perspective. Perspect Med Educ.

[14] Linn LS, Brook RH, Clark VA, Davies AR, Fink A, Kosecoff J (1985). Physician and patient satisfaction as factors related to the organization of internal medicine group practices. Med Care.

[15] Buchbinder SB, Wilson M, Melick CF, Powe NR (2001). Primary care physician job satisfaction and turnover. Am J Manag Care.

[16] Hall CB, Brazil K, Wakefield D, Lerer T, Tennen H (2010). Organizational culture, job satisfaction, and clinician turnover in primary care. J Prim Care Community Health.

[17] Hodkinson A, Zhou A, Johnson J (2022). Associations of physician burnout with career engagement and quality of patient care: systematic review and meta-analysis. BMJ.

[18] Williams ES, Skinner AC (2003). Outcomes of physician job satisfaction: a narrative review, implications, and directions for future research. Health Care Manage Rev.

[19] Zaresani A, Scott A (2020). Does digital health technology improve physicians’ job satisfaction and work-life balance? A cross-sectional national survey and regression analysis using an instrumental variable. BMJ Open.

[20] Sengupta A, Sarkar S, Bhattacherjee A (2024). The relationship between telemedicine tools and physician satisfaction, quality of care, and patient visits during the COVID-19 pandemic. Int J Med Inform.

[21] Lawrence K, Nov O, Mann D, Mandal S, Iturrate E, Wiesenfeld B (2022). The impact of telemedicine on physicians’ after-hours electronic health record “Work Outside Work” during the COVID-19 pandemic: retrospective cohort study. JMIR Med Inform.

[22] Leigh JP, Kravitz RL, Schembri M, Samuels SJ, Mobley S (2002). Physician career satisfaction across specialties. Arch Intern Med.

[23] Haleem A, Javaid M, Singh RP, Suman R (2021). Telemedicine for healthcare: capabilities, features, barriers, and applications. Sens Int.

[24] Grant AM (2007). Relational job design and the motivation to make a prosocial difference. AMR.

[25] Gkliati A, Saiti A (2022). Work engagement and job satisfaction in the medical sector: two aspects of psychological well-being among medical staff. Int J Health Sci.

[26] Bailey C, Yeoman R, Madden A, Thompson M, Kerridge G (2019). A review of the empirical literature on meaningful work: progress and research agenda. Hum Resour Dev Rev.

[27] Wanous JP, Reichers AE, Hudy MJ (1997). Overall job satisfaction: how good are single-item measures?. J Appl Psychol.

[28] Dolbier CL, Webster JA, McCalister KT, Mallon MW, Steinhardt MA (2005). Reliability and validity of a single-item measure of job satisfaction. Am J Health Promot.

[29] (2010). Quality of psychology test between Likert scale 5 and 6 points. J Soc Sci.

[30] Leung SO (2011). A comparison of psychometric properties and normality in 4-, 5-, 6-, and 11-Point Likert Scales. J Soc Serv Res.

[31] Van der Feltz-Cornelis CM, Van Oppen P, Van Marwijk HWJ, De Beurs E, Van Dyck R (2004). A patient-doctor relationship questionnaire (PDRQ-9) in primary care: development and psychometric evaluation. Gen Hosp Psychiatry.

[32] Long JS, Freese J (2014). Regression Models for Categorical Dependent Variables Using Stata.

[33] Williams R (2006). Generalized ordered logit/partial proportional odds models for ordinal dependent variables. Stata J.

[34] Breen R, Karlson KB, Holm A (2013). Total, direct, and indirect effects in logit and probit models. Sociol Methods Res.

[35] Karlson KB, Holm A, Breen R (2012). Comparing regression coefficients between same-sample nested models using logit and probit. Sociol Methodol.

[36] (2024). Majority of UK physicians see continued rise in telemedicine usage, reveals globaldata. GlobalData.

[37] Scott A, Bai T, Zhang Y (2021). Association between telehealth use and general practitioner characteristics during COVID-19: findings from a nationally representative survey of Australian doctors. BMJ Open.

[38] Pylypchuk Y, Barker W (2021). ASTP Health IT Data Brief.

[39] Hopkins L, Pedwell G (2021). The COVID PIVOT - re-orienting child and youth mental health care in the light of pandemic restrictions. Psychiatr Q.

[40] Stefos T, Carey K, Shen ML, Poe S, Oh DH, Moran E (2021). The effect of telehealth services on provider productivity. Med Care.

[41] Carrillo de Albornoz S, Sia KL, Harris A (2022). The effectiveness of teleconsultations in primary care: systematic review. Fam Pract.

[42] Mabeza RMS, Maynard K, Tarn DM (2022). Influence of synchronous primary care telemedicine versus in-person visits on diabetes, hypertension, and hyperlipidemia outcomes: a systematic review. BMC Prim Care.

[43] Guarino M, Cossiga V, Fiorentino A, Pontillo G, Morisco F (2020). Use of telemedicine for chronic liver disease at a single care center during the COVID-19 pandemic: prospective observational study. J Med Internet Res.

[44] Sandberg J, Trief PM, Izquierdo R (2009). A qualitative study of the experiences and satisfaction of direct telemedicine providers in diabetes case management. Telemed J E Health.

[45] Maria ARJ, Serra H, Castro MG, Heleno B (2022). Interaction at the primary-secondary care interface: patients’ and physicians’ perceptions of teleconsultations. Digit Health.

[46] Li T, Guan L, Zhang R (2023). Roles of doctor-patient relationship perception and job satisfaction in the impact of workplace violence on medical professionals’ turnover intentions in the early phase of COVID-19: a cross-sectional study in China. BMJ Open.

