# Supplementary material for: Association Between Telemedicine Adoption and Physician Job Satisfaction: Cross-Sectional Study
Source: J Med Internet Res. 2026 Mar 31;28:e82285. doi: 10.2196/82285 (PMC13038179; doi:10.2196/82285)
Supplement: Multimedia Appendix 1 [file jmir-v28-e82285-s001.docx]

**Association Between Telemedicine Adoption and Physician Job Satisfaction: Cross-Sectional Study**

**Multimedia Appendix**

Table S1. Brant test of parallel regression assumption

Table S2. Robustness check using ordinary least squares regression

Table S3. Robustness check with alternative job satisfaction measure (5-point Likert item)

Table S4. Robustness check using weekly online patient volume as continuous telemedicine measure

Table S1 Brant test of parallel regression assumption

|  | *χ*^2^ | ***P*** | **df** |
| --- | --- | --- | --- |
| **All** | 357.04 | <.001 | 116 |
| **Telemedicine adoption (reference=no)** |  |  |  |
| Yes | 4.16 | .39 | 4 |
| **Sex (reference=female)** |  |  |  |
| Male | 15.60 | .004 | 4 |
| **Age (reference ≤30)** |  |  |  |
| 31~44 | 0.26 | .99 | 4 |
| 45~59 | 3.64 | .46 | 4 |
| ≥60 | 0.63 | .96 | 4 |
| **Marital status (reference=unmarried)** |  |  |  |
| Married | 22.32 | <.001 | 4 |
| **Education (reference=associate degree or below)** |  |  |  |
| Bachelor’s degree | 2.54 | .64 | 4 |
| Master’s degree | 5.39 | .25 | 4 |
| Doctoral degree | 6.63 | .16 | 4 |
| **Professional titles (reference=no title)** |  |  |  |
| Junior | 6.08 | .19 | 4 |
| Mid-level | 6.03 | .20 | 4 |
| Senior | 6.21 | .18 | 4 |
| **Hospital type (reference=tertiary hospitals)** |  |  |  |
| Secondary hospitals | 0.72 | .95 | 4 |
| Primary hospitals | 0.78 | .94 | 4 |
| Private hospitals | 52.97 | <.001 | 4 |
| **Employment status (reference=contract employee)** |  |  |  |
| Permanent employee | 3.43 | .49 | 4 |
| **Medical specialty (reference=clinical)** |  |  |  |
| Medical technology | 6.26 | .18 | 4 |
| General practitioner | 1.56 | .82 | 4 |
| Others | 1.81 | .77 | 4 |
| **Managerial position (reference=no)** |  |  |  |
| Yes | 5.98 | .20 | 4 |
| **Years in practice** | 5.88 | .21 | 4 |
| **Years of service at the current institution** | 5.12 | .28 | 4 |
| **ln(Income)** | 2.62 | .62 | 4 |
| **Weekly working hours** | 4.55 | .34 | 4 |
| **Work stress (reference=low)** |  |  |  |
| Moderate | 289.21 | <.001 | 4 |
| High | 64.37 | <.001 | 4 |
| **Depression (reference=no)** |  |  |  |
| Yes | 15.00 | .005 | 4 |
| **Anxiety (reference=no)** |  |  |  |
| Yes | 18.16 | .001 | 4 |
| **Physician-patient relationship** | 67.04 | <.001 | 4 |

Table S2 Robustness check using ordinary least squares regression

| **Variables** | **Physician job satisfaction** | | |
| --- | --- | --- | --- |
|  | **Coefficient** | **95% CI** | ***P*** |
| **Telemedicine adoption (reference=no)** |  |  |  |
| Yes | 0.09 | 0.03 to 0.15 | .006 |
| **Sex (reference=female)** |  |  |  |
| Male | 0.06 | 0.02 to 0.11 | .003 |
| **Age (reference ≤30)** |  |  |  |
| 31~44 | −0.03 | −0.10 to 0.04 | .44 |
| 45~59 | 0.06 | −0.06 to 0.18 | .31 |
| ≥60 | 0.21 | −0.01 to 0.43 | .07 |
| **Marital status (reference=unmarried)** |  |  |  |
| Married | 0.11 | 0.05 to 0.17 | <.001 |
| **Education (reference=associate degree or below)** |  |  |  |
| Bachelor’s degree | −0.12 | −0.20 to −0.03 | .01 |
| Master’s degree | −0.17 | −0.28 to −0.06 | .002 |
| Doctoral degree | −0.20 | −0.34 to −0.07 | .003 |
| **Professional titles (reference=no title)** |  |  |  |
| Junior | −0.15 | −0.30 to −0.01 | .04 |
| Mid-level | −0.33 | −0.48 to −0.18 | <.001 |
| Senior | −0.31 | −0.47 to −0.15 | <.001 |
| **Hospital type (reference=tertiary hospitals)** |  |  |  |
| Secondary hospitals | −0.11 | −0.17 to −0.05 | <.001 |
| Primary hospitals | −0.17 | −0.25 to −0.09 | <.001 |
| Private hospitals | 0.09 | −0.24 to 0.41 | .61 |
| **Employment status (reference=contract employee)** |  |  |  |
| Permanent employee | −0.09 | −0.13 to −0.04 | <.001 |
| **Medical specialty (reference=clinical)** |  |  |  |
| Medical technology | 0.13 | 0.01 to 0.25 | .03 |
| General practitioner | 0.01 | −0.06 to 0.09 | .71 |
| Others | 0.03 | −0.01 to 0.08 | .14 |
| **Managerial position (reference=no)** |  |  |  |
| Yes | 0.21 | 0.16 to 0.26 | <.001 |
| **Years in practice** | 0.0003 | −0.01 to 0.01 | .91 |
| **Years of service at the current institution** | −0.004 | −0.01 to −0.0001 | .046 |
| **ln(Income)** | 0.02 | −0.01 to 0.04 | .20 |
| **Weekly working hours** | −0.003 | −0.004 to −0.001 | <.001 |
| **Work stress (reference=low)** |  |  |  |
| Moderate | −0.51 | −0.58 to −0.44 | <.001 |
| High | −0.45 | −0.52 to −0.37 | <.001 |
| **Depression (reference=no)** |  |  |  |
| Yes | −0.35 | −0.46 to −0.24 | <.001 |
| **Anxiety (reference=no)** |  |  |  |
| Yes | −0.23 | −0.31 to −0.16 | <.001 |
| **Physician-patient relationship** | 0.65 | 0.61 to 0.68 | <.001 |

Table S3 Robustness check with alternative job satisfaction measure (5-point Likert item)

| **Variables** | **Physician job satisfaction** | | |
| --- | --- | --- | --- |
|  | **OR** | **95% CI** | ***P*** |
| **Telemedicine adoption (reference=no)** |  |  |  |
| Yes | 1.13 | 1.00 to 1.27 | .04 |
| **Sex (reference=female)** |  |  |  |
| Male | 0.95 | 0.88 to 1.03 | .25 |
| **Age (reference ≤30)** |  |  |  |
| 31~44 | 0.98 | 0.86 to 1.13 | .82 |
| 45~59 | 1.11 | 0.88 to 1.39 | .38 |
| ≥60 | 1.51 | 1.00 to 2.28 | .048 |
| **Marital status (reference=others)** |  |  |  |
| Married | 1.09 | 0.98 to 1.22 | .11 |
| **Education (reference=associate degree or below)** |  |  |  |
| Bachelor’s degree | 0.86 | 0.73 to 1.02 | .08 |
| Master’s degree | 0.81 | 0.65 to 0.99 | .04 |
| Doctoral degree | 0.77 | 0.60 to 0.99 | .04 |
| **Professional titles (reference=no title)** |  |  |  |
| Junior | 1.04 | 0.80 to 1.37 | .76 |
| Mid-level | 0.71 | 0.53 to 0.94 | .02 |
| Senior | 0.73 | 0.54 to 0.995 | .047 |
| **Hospital type (reference=tertiary hospitals)** |  |  |  |
| Secondary hospitals | 0.77 | 0.69 to 0.86 | <.001 |
| Primary hospitals | 0.61 | 0.53 to 0.71 | <.001 |
| Private hospitals | 0.58 | 0.30 to 1.10 | .09 |
| **Employment status (reference=contract employee)** |  |  |  |
| Permanent employee | 0.94 | 0.86 to 1.02 | .15 |
| **Medical specialty (reference=clinical)** |  |  |  |
| Medical technology | 1.23 | 0.995 to 1.53 | .06 |
| General practitioner | 1.02 | 0.88 to 1.17 | .81 |
| Others | 1.03 | 0.94 to 1.12 | .53 |
| **Managerial position (reference=no)** |  |  |  |
| Yes | 1.68 | 1.52 to 1.86 | <.001 |
| **Years in practice** | 1.01 | 0.997 to 1.02 | .16 |
| **Years of service at the current institution** | 0.99 | 0.98 to 0.997 | .003 |
| **ln(Income)** | 1.12 | 1.06 to 1.18 | <.001 |
| **Weekly working hours** | 0.99 | 0.988 to 0.992 | <.001 |
| **Work stress (reference=low)** |  |  |  |
| Moderate | 0.38 | 0.33 to 0.44 | <.001 |
| High | 0.27 | 0.23 to 0.31 | <.001 |
| **Depression (reference=no)** |  |  |  |
| Yes | 0.55 | 0.45 to 0.67 | <.001 |
| **Anxiety (reference=no)** |  |  |  |
| Yes | 0.59 | 0.51 to 0.67 | <.001 |
| **Physician-patient relationship** | 3.53 | 3.27 to 3.79 | <.001 |

Table S4 Robustness check using weekly online patient volume as continuous telemedicine measure

| **Variables** | **Physician job satisfaction** | | |
| --- | --- | --- | --- |
|  | **OR** | **95% CI** | ***P*** |
| **Weekly online patients** | 1.01 | 1.001 to 1.01 | .01 |
| **Sex (reference=female)** |  |  |  |
| Male | 1.16 | 1.08 to 1.25 | <.001 |
| **Age (reference ≤30)** |  |  |  |
| 31~44 | 0.97 | 0.85 to 1.10 | .59 |
| 45~59 | 1.11 | 0.90 to 1.37 | .34 |
| ≥60 | 1.44 | 0.98 to 2.11 | .07 |
| **Marital status (reference=others)** |  |  |  |
| Married | 1.14 | 1.03 to 1.27 | .01 |
| **Education (reference=associate degree or below)** |  |  |  |
| Bachelor’s degree | 0.79 | 0.68 to 0.93 | .003 |
| Master’s degree | 0.73 | 0.60 to 0.88 | .001 |
| Doctoral degree | 0.73 | 0.58 to 0.92 | .007 |
| **Professional titles (reference=no title)** |  |  |  |
| Junior | 0.83 | 0.64 to 1.06 | .14 |
| Mid-level | 0.61 | 0.47 to 0.80 | <.001 |
| Senior | 0.62 | 0.46 to 0.82 | .001 |
| **Hospital type (reference=tertiary hospitals)** |  |  |  |
| Secondary hospitals | 0.82 | 0.74 to 0.91 | <.001 |
| Primary hospitals | 0.70 | 0.61 to 0.80 | <.001 |
| Private hospitals | 1.59 | 0.86 to 2.92 | .14 |
| **Employment status (reference=contract employee)** |  |  |  |
| Permanent employee | 0.86 | 0.79 to 0.93 | <.001 |
| **Medical specialty (reference=clinical)** |  |  |  |
| Medical technology | 1.22 | 1.00 to 1.49 | .05 |
| General practitioner | 1.02 | 0.89 to 1.16 | .78 |
| Others | 1.04 | 0.96 to 1.13 | .29 |
| **Managerial position (reference=no)** |  |  |  |
| Yes | 1.46 | 1.33 to 1.60 | <.001 |
| **Years in practice** | 1.003 | 0.99 to 1.01 | .53 |
| **Years of service at the current institution** | 0.99 | 0.99 to 1.00 | .07 |
| **ln(Income)** | 1.02 | 0.97 to 1.07 | .37 |
| **Weekly working hours** | 0.996 | 0.994 to 0.998 | <.001 |
| **Work stress (reference=low)** |  |  |  |
| Moderate | 0.27 | 0.24 to 0.31 | <.001 |
| High | 0.34 | 0.29 to 0.39 | <.001 |
| **Depression (reference=no)** |  |  |  |
| Yes | 0.60 | 0.50 to 0.73 | <.001 |
| **Anxiety (reference=no)** |  |  |  |
| Yes | 0.69 | 0.61 to 0.79 | <.001 |
| **Physician-patient relationship** | 3.51 | 3.27 to 3.76 | <.001 |
